# D-sorbitol-induced phase control of TiO_2_ nanoparticles and its application for dye-sensitized solar cells

**DOI:** 10.1038/srep20103

**Published:** 2016-02-09

**Authors:** Shoyebmohamad F. Shaikh, Rajaram S. Mane, Byoung Koun Min, Yun Jeong Hwang, Oh-shim Joo

**Affiliations:** 1 Clean Energy Research Center, Korea Institute of Science and Technology, Hawolgok-dong, Seongbuk-gu, Seoul, 136-791, Republic of Korea; 2 Department of Clean Energy and Chemical Engineering, Korea University of Science and Technology, Daejeon, 305-350, Republic of Korea; 3 School of Physical Sciences, Swami Ramanand Teerth Marathwada University, Nanded, 431 606, India; 4 Green School, Korea University, Anam-dong Seongbuk-gu, Seoul, 136-713, Republic of Korea

## Abstract

Using a simple hydrothermal synthesis, the crystal structure of TiO_2_ nanoparticles was controlled from rutile to anatase using a sugar alcohol, D-sorbitol. Adding small amounts of D-sorbitol to an aqueous TiCl_4_ solution resulted in changes in the crystal phase, particle size, and surface area by affecting the hydrolysis rate of TiCl_4_. These changes led to improvements of the solar-to-electrical power conversion efficiency (η) of dye-sensitized solar cells (DSSC) fabricated using these nanoparticles. A postulated reaction mechanism concerning the role of D-sorbitol in the formation of rutile and anatase was proposed. Fourier-transform infrared spectroscopy, ^13^C NMR spectroscopy, and dynamic light scattering analyses were used to better understand the interaction between the Ti precursor and D-sorbitol. The crystal phase and size of the synthesized TiO_2_ nanocrystallites as well as photovoltaic performance of the DSSC were examined using X-ray diffraction, Raman spectroscopy, field-emission scanning electron microscopy, high-resolution transmission electron microscopy, and photocurrent density-applied voltage spectroscopy measurement techniques. The DSSC fabricated using the anatase TiO_2_ nanoparticles synthesized in the presence of D-sorbitol, exhibited an enhanced η (6%, 1.5-fold improvement) compared with the device fabricated using the rutile TiO_2_ synthesized without D-sorbitol.

Titanium dioxide (TiO_2_) deserves special attention because of its non-toxicity, facile preparation with diverse morphologies, stability in both acidic and alkaline media, and wide applications for energy storage devices and photocatalysts^1^,^2^. The best-known TiO_2_ crystal structures are (in order of abundance) rutile, anatase, and brookite^3^, and the uniqueness of each lattice structure leads to multifaceted physicochemical and optoelectronic properties. These properties yield different functionalities, thus influencing their performances in various applications^4^. For instance, rutile phase of TiO_2_ exhibits a high refractive index and high UV absorptivity and is thus capable of being applied in optical communication devices (isolators, modulators, and switches etc.). Meanwhile, anatase is largely preferred in photovoltaics and photocatalysis because of its superior electron mobility and catalytic activity compared with the other two phases^5^. Beyond the crystal structures, these applications also require control of both the size and shape (or the facets exposed on the surface) of the nanostructures.

The phase transition of TiO_2_ polymorphs is an active area of research from the viewpoints of scientific interest and technological applications. Because there is no equilibrium temperature between the polymorphs of TiO_2_, a specific temperature regime for the occurrence of the phase transition is not yet been well-defined and explored^6^. It is accepted that the phase transformation pathways are affected by various intrinsic parameters (i.e., particle size, phase purity, nature of the Ti precursor, surface energy, density of defects, aggregation tendency, and crystal growth dynamics etc.) coupled with external factors including peptization, addition of modifiers/surfactant/chelating agents, and annealing ambience etc.^7^,^8^. Therefore, the development of facile and low-temperature solution-based methods to prepare crystalline TiO_2_ with tunable phase/size/morphology has opened a grand research avenue^9^.

Many solution based methods have been reported for the synthesis of TiO_2_ nanoparticles, such as sol–gel^10^, solvothermal^11^, and hydrolysis^12^ etc. Among these, hydrothermal synthesis method has the advantages of providing mono-dispersed particles, controlled structural morphology, and phase homogeneity etc., at relatively low temperatures^13^. In the hydrothermal synthesis, various parameters are being suggested to affect the crystallinity as well as the size of the TiO_2_ product. Zheng *et al.* proposed a dissolution-precipitation mechanism for TiO_2_ formation in which the concentration of the TiCl_4_ precursor was considered to determine the crystallinity of the TiO_2_ product; anatase crystallites grew larger and transformed into rutile^14^. The thermodynamic stability is reported to depend on the particle size, and anatase phase of TiO_2_ is more stable than rutile phase at particle diameters below approximately 14 nm^15^. In addition, the pH of the precursor solutions was also suggested to affect the growth mechanisms and thus crystal structures of the TiO_2_ nanocrystals^16^. The acidic/alkaline conditions employed in the synthesis of TiO_2_ nanoparticles were observed to affect the performance of DSSC^17^. However, determining how to control the conditions necessary to yield TiO_2_ nanocrystals with a definite crystal shapes and surface orientations to meet the requirements of DSSC remains a crucial problem.

The aim of the present work is to prepare TiO_2_ nanocrystals with pure anatase phase using a low-temperature (<200 °C) hydrothermal method. We investigated the role of D-sorbitol as a complexing agent on the formation of anatase TiO_2_. D-sorbitol was selected because of its non-toxic biological origin and environmentally friendly nature, low cost, and ability to assist complex formation. In the present study, we observed that reactions proceeds even in the absence of D-sorbitol however, the resultant TiO_2_ product was pure rutile rather than anatase. The driving force for the anatase TiO_2_ synthesis was studied from the complex species of D-sorbitol with Ti cations coupled through hydroxyl ions in the solution. The solution approach offered the possibility to control the reaction pathways on a molecular level and enabled the synthesis with a well-defined crystal polymorph and morphologies without impurities. The effects of the preparation conditions on the crystal phase of the TiO_2_ nanocrystals as well as the photovoltaic performance of DSSC equipped with the prepared TiO_2_ nanocrystals were studied. It was observed that the nanocrystallites of anatase TiO_2_ prepared using the hydrothermal method exhibited comparable/enhanced DSSC performance compared with the commercial anatase/rutile TiO_2_. The present method is a facile single-step process, and TiO_2_ nanoparticles prepared in this present work are chemically, environmentally and mechanically stable for several days, justifying their long-term uses.

## Results and Discussion

### Reaction mechanism

In context with the report of Gopal *et al.,* the experimental Ti–O phase diagram indicates that anatase is more stable than rutile at room temperature and atmospheric pressure^18^. Both anatase and rutile TiO_2_ consist of TiO_6_^2−^ octahedra, which share edges and corners in different manners. In the rutile case, two opposite edges of each octahedron are linked through a corner oxygen atom, forming linear chains of octahedra. In contrast, anatase exhibits no corner sharing but instead has four edges shared per octahedron. The anatase structure can be viewed as zigzag chains of TiO_6_^2−^ octahedral, linked to each other through edge-sharing bonding^19^. Because anatase has more edge sharing, and the interstitial spaces between octahedra are larger, it is less dense than rutile (the densities of rutile and anatase are 4.26 and 3.84 gcm^−3^, respectively)^20^. It has been accepted that when the four-fold Ti precursor ([TiCl_4_] or [Ti(ROH)_4_]) reacts with water, the coordination number of Ti^4+^ increases from four to six through its vacant *d*-orbitals to accept oxygen lone pairs from nucleophilic ligands^21^. These six-fold structural units undergo condensation and become the octahedra that are incorporated into the final precipitate structure. The octahedra agglomerate through corner and edge sharing during the condensation reactions^22^. During the particle agglomeration, the acidity of the reaction medium is suggested as a critical factor for the hydrolysis of TiCl_4_ in aqueous solution^23^. Under highly acidic conditions, the agglomeration of rutile TiO_2_ could be attributed to hydrogen bonding among the protonated nanocrystallites^24^. In addition, because of the lower surface energy of anatase compared with that of rutile, selective formation of the anatase phase is favored under weak acid conditions as polycondensation of Ti(OH)_n_Cl_6-n_ species is weak (slow)^25^. Cheng *et al.* also explained the difference in the crystallization of anatase and rutile TiO_2_ by the hydrolysis of TiCl_4_ in an aqueous solution using ligand field theory^26^, and the crystallization occurred *via* dehydration between partially hydrolyzed Ti(OH)_n_Cl_6-n_ complexes.

**Figure 1:**
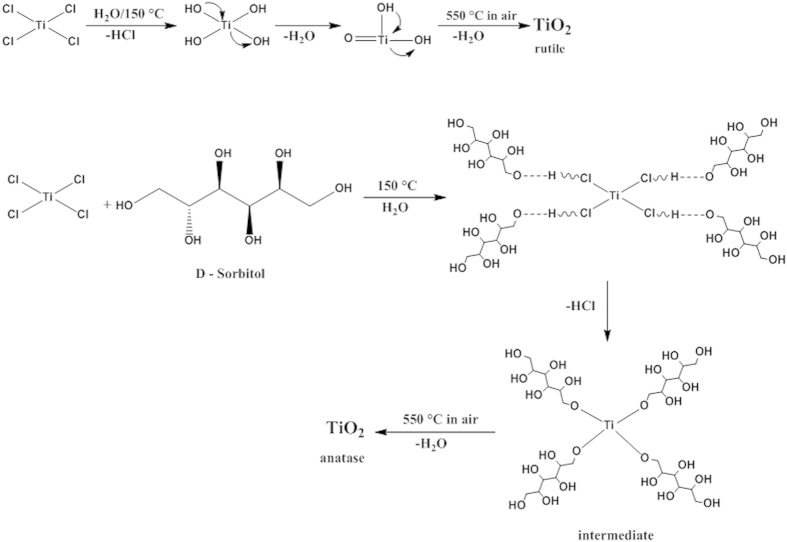
Reaction mechanism for the formation of anatase TiO_2_.

Adapting the previous studies, in the present study, the reaction mechanisms illustrated in Fig. 1 is proposed depending on the presence of D-sorbitol. When the effect of D-sorbitol on crystallization was considered, one can suppose that D-sorbitol anions substitute for the chlorine anions during the hydrolysis process to form [Ti(OH)_x_(D-sorbitol)_y_Cl_z_]^n−^ intermediate complexes, where 4 ≤ x + y + z ≤ 6. The detailed effect of D-sorbitol on the nucleation process of TiO_2_ is worthy of future investigation. Furthermore, the presence of HCl in the TiCl_4_ aqueous solution is expected to catalyze not only the nucleation of anatase TiO_2_ but also the crystal growth *via* condensation of the intermediate complexes and related species^27^.

### Study of intermediate complex formation

**Figure 2:**
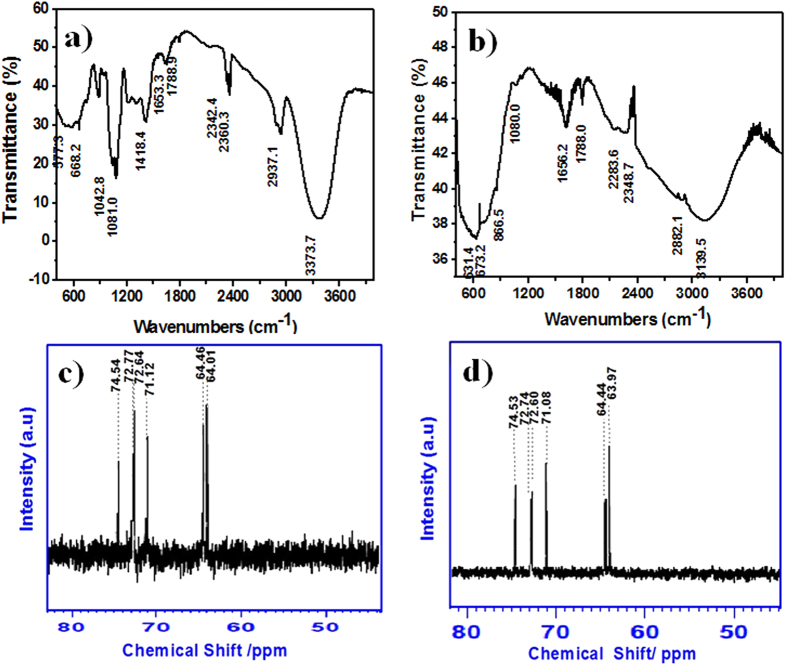
FT-IR spectra powder pellet of; (**a**) pure D-sorbitol and (**b**) an intermediate complex of TiO_2_–D-sorbitol. ^13^C NMR spectra of (**c**) 0.1M D-sorbitol in aqueous solution of pH** = **0.6 and (**d**) 1M TiCl_4_–0.1M D-sorbitol in aqueous solution of pH** = **0.6. D_2_O was used as an external standard.

To examine the proposed intermediate complex formation, FT-IR and ^13^C NMR spectra analyses of anatase TiO_2_ were attempted. After completion of the hydrothermal reaction between TiCl_4_ and D-sorbitol, we collected an intermediate product for FT-IR measurement. The dried intermediate product (0.2%) was mixed with KBr powder, and a pure KBr pellet was used as the baseline measurement. The FT-IR spectrum of pure D-sorbitol (Fig. 2a) contains strong peaks of –OH and –C–O stretching vibrations at 3373 and 1081 cm^−1^, respectively^28^, and the peak of C–H stretching vibration appears at 2937 cm^−1^. Several peaks of –C–H bending vibrations are observed between 1250 and 1418 cm^−1^ 29. Figure 2b shows the intermediate complex between Ti^4+^ and D-sorbitol ions. The D-sorbitol peak overlaps the intermediate complex, confirming the formation of a complex structure. Because of the molecular interaction of D-sorbitol with Ti ions, a small positive and negative peak shift is achieved. In the intermediate complex of TiO_2_-D-sorbitol, the broad peak at 3139.5 cm^−1^ corresponds to –OH stretching vibrations is evidenced. The broader nature of –OH stretching vibrations confirmed the presence of hydroxyl groups over different sites as well as the varying interaction between hydroxyl groups on anatase TiO_2_^30^. The high-intensity peak near 600 cm^−1^ is assigned to TiO_2_^31^.

For more confirmation of complex formation, we conducted ^13^C NMR spectroscopy measurements over pure D-sorbitol and TiCl_4_-D-sorbitol using D_2_O as a reference solution. The ^13^C NMR spectra were used to study the interaction of the metal and D-sorbitol complexes. Figure 2c,d highlight the peak values of six signals expected for coordinated D-sorbitol, which are similar to the elsewhere reported values^32^. The complex solution of TiCl_4_ and D-sorbitol shows chemically minute shifting of peak positions toward the upward direction, which indicates the formation of an intermediate complex.

### Surface morphology and structural analysis

**Figure 3:**
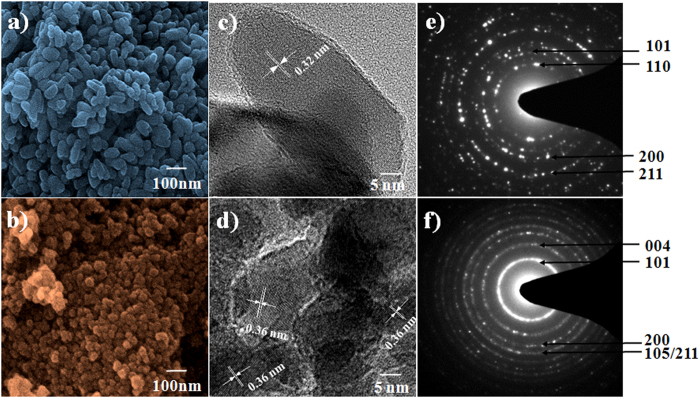
FE-SEM images of; (**a**) rutile-TiO_2_ and (b) anatase-TiO_2_; HR-TEM images of (c) rutile-TiO_2_ and (d) anatase-TiO_2_, and SAED patterns of; (e) rutile-TiO_2_ and (f) anatase-TiO_2_. Rutile-TiO_2_ was obtained when D-sorbitol was not used, whereas anatase-TiO_2_ was obtained when D-sorbitol was used.

Next, the morphology and crystal structure of the synthesized TiO_2_ nanocrystals were compared depending on the usage of D-sorbitol. The FE-SEM images demonstrate the uniformity of the synthesized TiO_2_ consisting of well-interconnected nanocrystallites. The average diameter decreased from ~60 nm to ~20 nm when D-sorbitol was present in the precursor solution (Fig. 3a,b), signifying capping capability of D-sorbital for an over growth. We observed a consistent size difference in the HR-TEM and BET data (see section below). In addition, the HR-TEM images of the as-prepared TiO_2_ nanocrystallites confirm their high crystallinity regardless of the presence of D-sorbitol in the precursor solutions. However, the measured lattice parameters for the TiO_2_ nanocrystallites changed, implying that different crystal phases were synthesized. The lattice parameters were measured from the HR-TEM images (Fig. 3c,d) and the positions of the main diffraction peaks in the SAED patterns (Fig. 3e,f). The distances between two adjacent lattice planes, for two cases, were 0.32 nm [in good agreement with the (110) crystallographic plane of rutile (Fig. 3c,e)], and 0.36 nm [in good agreement with that of (101) for anatase TiO_2_ (Fig. 3d,f)] (hence forth, called rutile TiO_2_ and anatase TiO_2_, respectively)^33^. The (101) crystal faces of anatase have lower surface energy and are expected to be more stable than the other faces^34^, and our HR-TEM images also demonstrated the strongest ring pattern of (101) in SAED spectrum.

**Figure 4:**
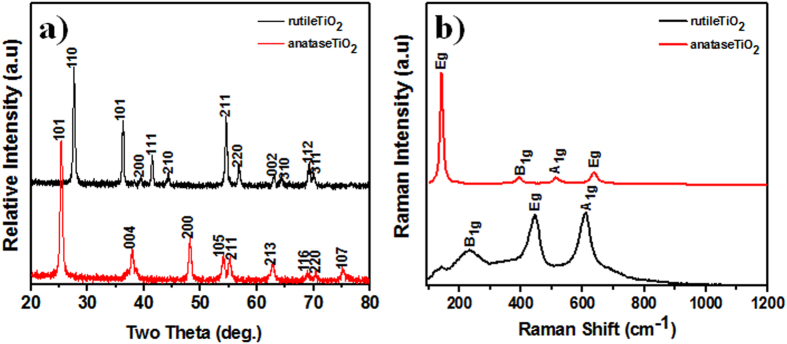
(**a**) XRD, and (**b**) Raman spectra of rutile and anatase TiO_2_ nanocrystallites.

The change in the crystal structures of the synthesized TiO_2_ nanocrystals was also confirmed by XRD patterns (Fig. 4a) and Raman spectrum (Fig. 4b), both of which consistently demonstrated that in the presence of D-sorbitol only anatase TiO_2_ is obtained; otherwise, rutile is favored. The observed XRD peaks were well attributed to rutile TiO_2_ (JCPDS no. 870710) and anatase TiO_2_ (JCPDS no. 86-1156). The observation of strong XRD peaks was indicative of the good crystallinity of the as-prepared TiO_2_. In table 1, crystal size, calculated using Scherrer formula, is presented. As-prepared anatase TiO_2_ nanocrystals were stable up to 700 °C and transformed into rutile as the calcination temperature increased to 800–1000 °C [Fig. S1a–d in the electronic supplementary information (ESI)]. These results indicate that compared with anatase, rutile is the thermodynamically stable phase of TiO_2_. Raman spectroscopy also corroborated the presence the rutile and anatase phases of TiO_2_. In Fig. 4b, the Raman shifts at 143, 235, 447, and 612 cm^−1^ are attributed to the B_1g_, two-phonon scattering, E_g_, and A_1g_ modes of the rutile phase, respectively^35^. The four Raman shift peaks at 144, 400, 514, and 638 cm^−1^ are attributed to the E_g_, B_1g_, A_1g_, and E_g_ symmetries of the anatase phase, respectively^36^.

In addition, as presented in Fig. S2(a–c), XPS analysis was used to investigate the chemical Ti^4+^ state of both the rutile and anatase TiO_2_ phases; more or less similar electronic states and chemical compositions were observed on the surface. Regardless of the preparation procedure (with or without D-sorbitol), the amounts of the adsorbed residues on the two TiO_2_ surfaces were similar.

### Hydrolysis rate estimation

**Figure 5:**
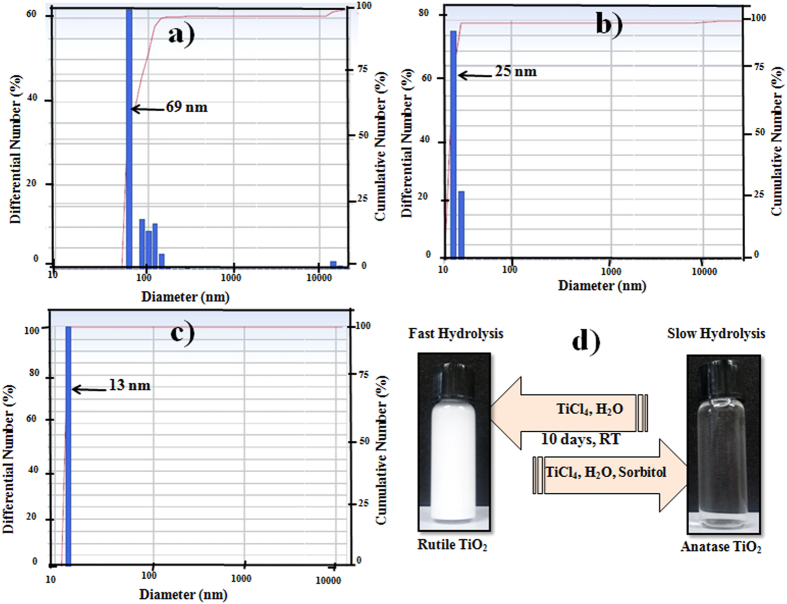
DLS spectra of; (**a**) 1M TiCl_4_ in aqueous solution, (**b**) TiCl_4_–0.05MD-sorbitol, and (**c**) TiCl_4_-0.1M D-sorbitol in aqueous solution and (**d**) diagram of room-temperature hydrolysis reaction of TiO_2_.

The dynamic light scattering (DLS) technique was used to study the effect of D-sorbitol on the hydrolysis process of TiCl_4_ at room temperature. The acidity (pH = 0.6) of the solutions for the DLS measurements was the same as those of the starting TiCl_4_ (1 M) and D-sorbitol solution to validate the comparisons. The resolution of the DLS apparatus was 2 nm. As observed in Fig. 5a, in the absence of D-sorbitol, TiCl_4_ in aqueous solution could hydrolyte to form particle agglomeration with a size distribution of ~69 nm. While in the presence of 0.05 M D-sorbitol the size distribution was ~25 nm, as observed in Fig. 5b. Moreover, Fig. 5c shows that for 0.1M D-sorbitol, the size distribution is decreased to 13 nm. With a further increase in the D-sorbitol concentration up to 0.15 M, no particle formation occurs. The systematic decrease in particle-size is an indication of an agglomeration-free reaction, supporting the conclusion that the interaction of D-sorbitol with TiCl_4_ prevents the rapid hydrolysis of TiCl_4_. The formation of small-sized anatase nanocrystallites as embryos could be due to the inhibition of crystal growth by the coordination of D-sorbitol anions. Consistent results were obtained by Ambade *et al.* for ZnO nanorods^37^.

To better understand the hydrolysis reaction, we kept both samples (TiCl_4_ solution in aqueous medium and TiCl_4_–0.1 M D-sorbitol) at room temperature for more than ten days (Fig. 5d). The TiCl_4_ solution in aqueous medium started to become turbid (white) with particles as sediment after two days. These primary crystallites subsequently coalesced, and a precipitate settled slowly. However, despite ~13 nm particle-size, the TiCl_4_-0.1 M D-sorbitol solution was clear and transparent until more than one month. This conclusion was also supported by the DLS measurement, where the D-sorbitol anion could bond to Ti cations by preventing the fast hydrolysis at room temperature. It is believed that the slow hydrolysis (as the solution is clear and transparent) plays a critical role in developing small-sized particles, which eventually can help in the phase transformation process from rutile to anatase. However, to obtain anatase TiO_2_ from the D-sorbitol-containing solution, an adequate temperature is necessary to initiate the nucleation process followed hydrolysis^38^.

**Figure 6:**
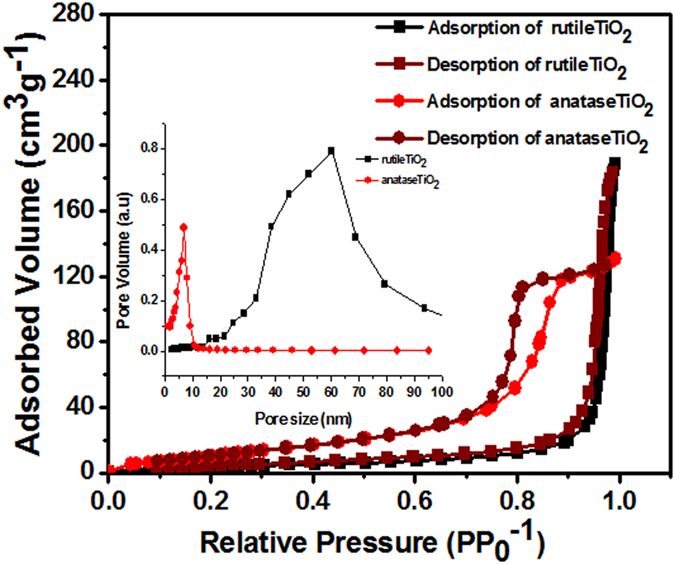
BET analysis (the inset shows the pore size distribution of TiO_2_ nanocrystallites).

Surface area and pore-size analysis. The specific surface area and pore-size distribution of both as-prepared TiO_2_ nanostructures were characterized using nitrogen gas adsorption. A type-IV isotherm and H1-type hysteresis loop were confirmed for both TiO_2_ nanostructures (Fig. 6), suggesting macroporosity in rutile and mesoporosity in anatase TiO_2_^39^. The specific surface area, calculated using the standard multi-point BET method, was 14.28 m^2^g^−1^ for rutile TiO_2_, which was only one-third to that of the anatase TiO_2_ (47.77 m^2^g^−1^). The as-prepared TiO_2_ exhibited a narrow pore-size distribution centered at 60.28 nm for rutile TiO_2_ and 16.79 nm for anatase TiO_2_ (inset of Fig. 6). The performance of DSSC depends on the type of porosity, particle/pore size and charge transport properties of the TiO_2_ photoanode^40^. Generally, smaller nanoparticles have a larger surface area but a shorter electron diffusion length, whereas larger nanoparticles have a longer electron diffusion length but a smaller surface area^41^. Because of the multiple factors, an optimal particle-size is required to achieve high solar-to-electrical power conversion efficiency (η). For example, Cao *et al.* concluded that a particle-size of 15 nm can be the best among 10–20 nm sized samples for superior DSSC application^42^.

### DSSC performance

**Figure 7:**
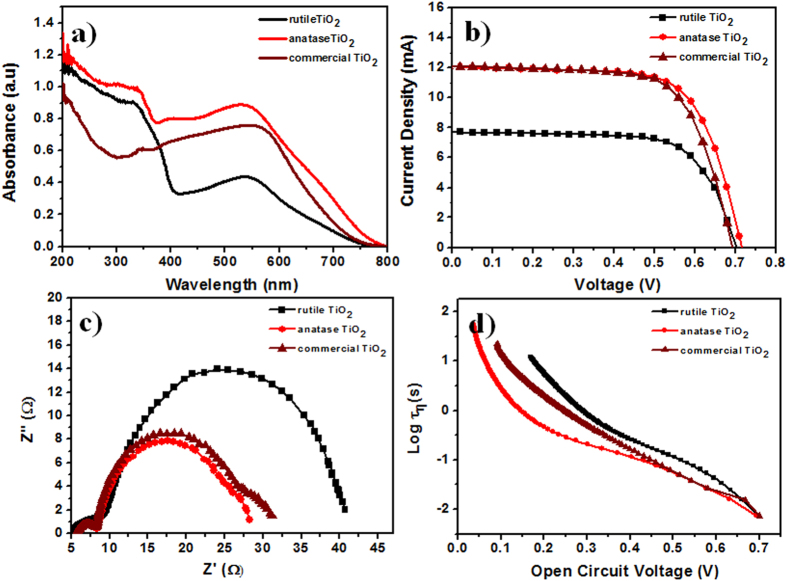
(**a**) UV–Vis, (**b**) J–V curve, (**c**) EIS, and (**d**) τ (*vs.*V_oc_) measurements of various TiO_2_ nanocrystal DSSC-photoanodes.

To understand the DSSC performance depending on the preparation methods, we first measured UV–Vis absorption spectra of dye-adsorbed photoanodes (Fig. 7a). All of the photoanodes exhibited a wide absorbance in the visible region (centered at approximately 530 nm); however, the prepared anatase TiO_2_ photoanode exhibited higher absorption compared with the commercial (100% anatase, for more details please see Experimental section) and rutile TiO_2_ electrodes, which is consistent with the order of the dye adsorption amounts on the TiO_2_ surfaces (Table 1). The different crystallinity, smaller particle-size, and higher surface area of the prepared anatase TiO_2_ could increase the dye adsorption, which is evident from the enhanced UV–Vis absorption. The performance of the DSSC was tested under illumination of simulated AM1.5 G solar light (100 mW cm^−2^), and the J-V characteristics are presented in Fig. 7b for each individual cell. In Table 1, the crystal phase and photovoltaic performance parameters are summarized. The short-circuit current density (J_SC_) of the anatase TiO_2_ photoanode (12.19 mA cm^−2^) was 1.5 times greater than that of the rutile TiO_2_ electrode (7.96 mA cm^−2^). In addition, the V_oc_ of the anatase TiO_2_ electrode was similar but increased by 0.02 V compared with that of the rutile electrode. Therefore, the η of the cells made of anatase TiO_2_ was 1.5 times higher (η = 6%) than that for rutile TiO_2_ (η = 3.8%), which is mainly attributed to the enhancement of J_SC_. The standard deviation of the photovoltaic parameters was calculated to validate the accuracy and reproducibility of the DSSC performance of the TiO_2_ nanocrystallites (Fig. S3). The remarkable performance of the DSSC fabricated with the anatase TiO_2_ electrode might originate from its crystal phase, morphology, and high electrical conductivity and mobility (Table S1)^43^. Contrary, due to the presence of several stacking faults and dislocations, electrode with rutile TiO_2_ nanocrysyallites demonstrated lower conductivity and low dye intake capacity and thereby, smaller light harvesting capacity and lower DSSC performance^44^. Generally, smaller particles provide more active sites for dye adsorption and reaction in DSSC because of the larger specific area, leading to higher photo-to-electric power conversion efficiency. Moreover, upon comparison with the commercial TiO_2_ electrode we observed that the crystal phase is a critical factor to achieve enhanced *η* for DSSC, which again indicates the importance of crystal phase control in TiO_2_ synthesis. Our preparation method revealed that D-sorbitol can successfully control the crystal phase of TiO_2_ to achieve high performance of DSSC.

To further explore the effects of the properties of TiO_2_ photoanodes on the performance of corresponding DSSC, EIS measurements were performed. Fig. 7c presents Nyquist plots of the three cells (i.e., anatase, commercial, and rutile TiO_2_) measured at a forward bias of V_oc_. Two semicircles, including a small one at higher frequency and a large one at lower frequency, are observed in the plots. The small semicircle is assigned to the charge-transfer resistance (R_1_) and the capacitance (CPE_1_) at the platinum counter electrode/redox electrolyte interface, whereas the larger semicircle is attributed to the recombination resistance (R_2_) and chemical capacitance (CPE_2_) at the TiO_2_/dye/redox electrolyte interface^45^. Therefore, the size of the second semicircle (the value of R_2_) is very important to understand the changes in the photoanode. Large difference in the R_2_ values is observed between the rutile and anatase TiO_2_ photoanodes. The anatase TiO_2_ photoanode exhibited a smaller R_2_ value (19.7 Ω) than the rutile TiO_2_ photoanode (31.0 Ω), indicating faster (hole) generation and transport as well as a slower electron-hole recombination rate. The electron lifetime (τ) was calculated according to the equation τ** = **(1/2π*f*_max_), where *f*_max_ is the maximum frequency of the mid-frequency peak^46^. The τ values, estimated from Bode phase plots, were 1.59 × 10^−4^, 2.24 × 10^−4^, and 2.18 × 10^−4^ ms for rutile, anatase, and commercial anatase TiO_2_, respectively (Table S2). For anatase TiO_2_, the higher τ value was due to the reduced charge transfer resistance and decreased electron recombination, enabling more efficient electron transfer with an enhancement of the device performance.

In addition, the decay of V_oc_ was used to reflect the regression of the electron density in the conduction band of the photoanodes as it is widely used as a kinetic parameter, which contains useful information about the rate constant of the electron transfer process in DSSC^47^,^48^,^49^. The τ values (Fig. 7d) were calculated by fitting the photovoltage decay plots obtained from the V_oc_ decay measurements and by applying an equation developed by Bisquert *et al.*^50^. The higher τ value for the anatase TiO_2_ photoanode implied a lower charge recombination rate and improved electron transfer efficiency compared with commercial and rutile TiO_2_, which is consistent with the impedance results and leads to an improvement in the DSSC performance.

## Conclusion

During hydrothermal growth of TiO_2_, D-sorbitol was demonstrated to be a crystal-phase-controlling agent. As-prepared TiO_2_ had a rutile crystal phase when prepared *via* the hydrolysis of the TiCl_4_ precursor in an acidic environment, whereas pure anatase TiO_2_ was obtained when D-sorbitol was added into the precursor solution. The intermediate complex formation between Ti ions and D-sorbitol molecules was recorded using FT-IR and ^13^C NMR spectroscopy of anatase TiO_2_. The DLS measurements supported the conclusion that the interaction between D-sorbitol and TiCl_4_ prevents its rapid hydrolysis, resulting in the systematic decrease in the TiO_2_ particle-size as the concentration of D-sorbitol increased. We expect that the slow hydrolysis plays a critical role for small-size particle formation and assists in the anatase phase transformation. The photovoltaic performances of the rutile and anatase TiO_2_ polymorphs were compared. Solar-to-electrical power conversion efficiency of the DSSC fabricated using the pure anatase TiO_2_ electrode was 6.0%, which was 1.5 times higher than that prepared using the rutile TiO_2_ (3.7%) electrode prepared under the same experimental conditions and comparable (5.8%) to commercial TiO_2_. Our study demonstrated that comparable DSSC performance achieved for anatase TiO_2_ prepared using a simple hydrothermal method might arise from its phase, crystal-size, morphology, surface orientation, and high electrical conductivity and mobility.

## Experimental Section

### Chemicals

All the chemicals were of analytical grade and used without any further purification. Titanium (IV) chloride (99.9%) and D-sorbitol (>98%) were purchased from Sigma Aldrich. Commercial TiO_2_ paste was also purchased (ENB Korea, 100% anatase, ~20 nm particle-size). The fluorine-doped tin oxide (FTO) substrate (15 Ω, TEC 8, Pilkington glass) was cleaned with soap and successively sonicated in distilled water, acetone, and isopropanol for 20 min, respectively, followed by drying with nitrogen gas flow. N-719 dye (Ruthenium 535-bis TBA) and an electrolyte (Iodolyte AN-50) were purchased from Solaronix.

### Hydrothermal synthesis of TiO_2_ nanostructures

TiO_2_ nanostructures were prepared using a simple one-step hydrothermal method. In the standard experimental procedure for the synthesis of the anatase phase, 5 mL of 1 M TiCl_4_ and 2.5 g D-sorbitol, were mixed in 80 mL of deionized (DI) water (Milli-Q water; 18.2 MΩ.cm). The mixture was constantly stirring for 10 min before transferring into a 100-mL Teflon-lined stainless-steel autoclave. The autoclave was sealed and maintained at 150 °C for 24 h, followed by cooling to room temperature. The resulting yellowish-white product was centrifuged at 8000 rpm for 10 min and washed several times with deionized water and ethanol (1:1 volume ratio) to remove any undesired impurities. The product was heated at 550 °C for 1 h to obtain the white powder of TiO_2_. The same experimental conditions were applied for the synthesis of rutile TiO_2_ except D-sorbitol.

### Characterizations

The surface morphologies of both the rutile and anatase TiO_2_ nanostructures were examined using field-emission scanning electron microscopy (FE-SEM, Nova NanoSEM200-100 FEI) images. The phases of the TiO_2_ photoanodes were confirmed by X-ray diffraction (XRD) spectra (XRD-6000, Shimadzu, Japan) obtained at Cu-Kα radiation (λ = 0.1542 nm). Phase analysis was additionally performed using a Raman microscope (Renishaw, inVia Raman microscope, UK) to corroborate the formation of rutile and anatase TiO_2_ phases. The laser beam (λ = 532 nm) was focused using a lens to produce a spot on the photoanode. Fourier-transform infrared (FT-IR) spectroscopy was measured from 500 to 4000 cm^−1^ using an IR spectrometer (Nicolet iS10, Smart MIRacle, Thermo Scientific). The high resolution transmission electron microscopy (HR-TEM) and selected area electron diffraction (SAED) measurements were performed using a FEI TECNAI G2 20 S-TWIN equipped with a LaB6 cathode and a GATAN MS794 PCCD camera. The micrographs were obtained at an acceleration voltage of 200 kV. The powders of TiO_2_ nanocrystals were suspended in ethanolic solutions separately and dropped onto a Formvar/carbon, 200 mesh TH, copper grids before HRTEM measurements. X-ray photoelectron spectroscopy (XPS) spectra were acquired using a PHI 5000 Versa Probe (Ulvac-PHI) using a monochromatic Al Kα X-ray source (1486.6 eV). The data were collected from a spot-size of 100 × 100 μm^2^. The carbon 1s peak (284.6 eV) was used as a reference for internal calibration. The UV-Vis absorption spectra of dye-adsorbed TiO_2_ photoanodes were recorded using a Varian Cary 5000 spectrophotometer. To quantify the amounts of dye adsorbed onto the TiO_2_ photoanodes, the dye molecules were desorbed by dipping in 0.1 M NaOH solution (ethanol and water at a 1:1 ratio) for 24 h at room temperature. The specific surface area was measured using Brunauer–Emmett–Teller (BET) technique (Belsorp II, BEL Japan INC). The dynamic light scattering (DLS) technique (Photal Otsuka electronics ELSZ-1000 instrument) was used to understand the particle-size variation. ^13^CNMR spectra were measured (Bruker 400-MHz FT-NMR, D_2_O) with δ and values from large to small.

### Fabrication and evaluation of DSSC

TiO_2_ paste was prepared by mixing 1.0 g TiO_2_ powder, 3.5 g α-terpineol, and 0.5 g ethyl cellulosein ethanol (3.0 mL) and acetic acid (0.2 mL) solvent and stirring for 24 h to form homogeneous slurry, separately for each TiO_2_ phase. TiO_2_ colloid paste was spread over the FTO substrate *via* a doctor blade technique with adhesive tape as a spacer. The substrate was sintered at 450 °C for 30 min in air, which resulted in an approximately 10-μm-thick TiO_2_ porous film. The dye sensitizer used in this work was cis-di(isothiocyanato)-bis-(2,2-bipyridyl-4,4-dicarboxylato)ruthenium(II)bis-tetrabutyl ammonium (so-called N-719, 0.5 mM in a mixed solvent of acetonitrile and *tert*-butanol in a volume ratio of 1:1), which was used as received from Solaronix. DSSC were assembled by adding an electrolyte solution (0.6 M tetrapropyl ammonium iodide, 0.1 M iodine, 0.1 M lithium iodide, and 0.5 M 4-*tert*-butylpyridine in acetonitrile) between the dye-sensitized TiO_2_ photoanode and a platinized conducting-glass electrode. The two electrodes were clipped together, and a cyanoacrylate adhesive was used as a sealant to prevent leakage of the electrolyte solution. A solar simulator (150-W Xe lamp, Sun 2000 solar simulator, ABET 5 Technologies, USA) equipped with an A.M. 1.5G filter was used to generate simulated sunlight, and the intensity of 1 sun (100 mW cm^−2^) was calibrated with a reference silicon solar cell. The photocurrent density-applied voltage (J-V) spectra of various TiO_2_ photoanodes were obtained with the aid of a Keithley 2400 source meter. The electrochemical impedance spectroscopy (EIS) measurements of the TiO_2_ photoanodes were recorded using a two-electrode system by a potentiostat (IviumStat Technologies, Netherland) in the frequency ranges of 150 kHz to 0.1 Hz.

## Additional Information

**How to cite this article**: Shaikh, S. F. *et al.* D-sorbitol-induced phase control of TiO_2_ nano-particles and its application for dye-sensitized solar cells. *Sci. Rep.* **6**, 20103; doi: 10.1038/srep20103 (2016).

## Supplementary Material

Supplementary Information

## Figures and Tables

**Table 1:** DSSC parameters of various TiO_2_ nanocrystallite photoanodes.

Photoanode	Crystalsize (nm)	Dye Adsorption[×10^−7^mol cm^−2^]	J_sc_ (mA cm^−2^)	V_oc_ (V)	FF	η (%)
rutile TiO_2_	19.5	0.42	7.96	0.69	0.68	3.8
Anatase TiO_2_	15.1	0.95	12.19	0.71	0.69	6.0
Commercial anatase TiO_2_	16.2	0.81	12.25	0.70	0.65	5.8
