# Supplementary material for: D-sorbitol-induced phase control of TiO2 nanoparticles and its application for dye-sensitized solar cells
Source: Sci Rep. 2016 Feb 9;6:20103. doi: 10.1038/srep20103 (PMC4746568; doi:10.1038/srep20103)
Supplement: Supplementary Information [file srep20103-s1.docx]

**Supporting information**

**D-sorbitol-induced phase control of TiO_2_ nanoparticles and its application for dye-sensitized solar cells**

Shoyebmohamad F. Shaikh^1,2^, Rajaram S. Mane^3^, Byoung Koun Min^1,2,4^, Yun Jeong Hwang,^1,2*^ Oh-shim Joo^1,2*^

^1^Clean Energy Research Center, Korea Institute of Science and Technology, Hawolgok-dong, Seongbuk-gu, Seoul, 136-791, Republic of Korea

^2^Department of Clean Energy and Chemical Engineering, Korea University of Science and Technology, Daejeon 305-350, Republic of Korea

^3^School of Physical Sciences, Swami Ramanand Teerth Marathwada University, Nanded, 431606, India.

^4^Green School, Korea University, Anam-dong Seongbuk-gu, Seoul, 136-713, Republic of Korea

**Effect of thermal annealing on anatase-to-rutile Phase Transition**

Fig. S1 (a) was shown XRD patterns of the anatase TiO_2_ powder calcined at various temperature range from 550°C to 1000°C. The peak intensity at 550°C shown pure anatase TiO_2_ phase but after increased annealing temperature up to 800°C, mixed (anatase$+$rutile) phase formation take place. Moreover, intensity of anatase TiO_2_peak decrease while rutile TiO_2_ peak intensity increase. Furthermore, enhancement in annealing temperature up to 1000°C, the pure rutile TiO_2_ phase formation takes place. Same temperature variation was conducted in the case of rutile TiO_2_ powder as synthesized in the absence of D-sorbitol. It can be shown no phase variation after thermal treatment, implying rutile phase is thermodynamically stable phase compare with anatase TiO_2_.

Fig. S1 (b) was shown the scheme diagram of phase variation *Vs* annealing temperature. Thestabilities for the different shapes of TiO_2_ are confirmed by their anatase-to-rutile phase transition temperature. Fig. S1(c) shows the anatase-to-rutile transition curves for the two kinds of TiO_2_nanocrystallites. The percentage of rutile phase is calculated using XRD data and according to the following formula:

$$W_{R}\left( \% \right)= \frac{1}{1+0.8 (\frac{I_{A}}{I_{R}})}\times100$$

Where, W_R_ is the weight percentage of rutile in the TiO_2_ sample and I_A_ and I_R_ are the intensities of the anatase 101 and rutile 110 peaks, respectively. It can show that up to increased annealing temperature at 750°C, the anatase phase of TiO_2_ is stable and not change to rutile TiO_2_. When the sample was calcined at 800°C, the intensities of the rutile peak increased, but also small peak intensity of anatase phase was still there. That was confirming the mixed phase formation take placeat 800°C. Moreover, annealing temperature increased up to 1000°C the characteristic peak of anatase phase disappears and pure rutile phase was observed. Fig. S1 (d) showed that systematic increase annealing temperature affected the crystal size of TiO_2_nanocrystallite.


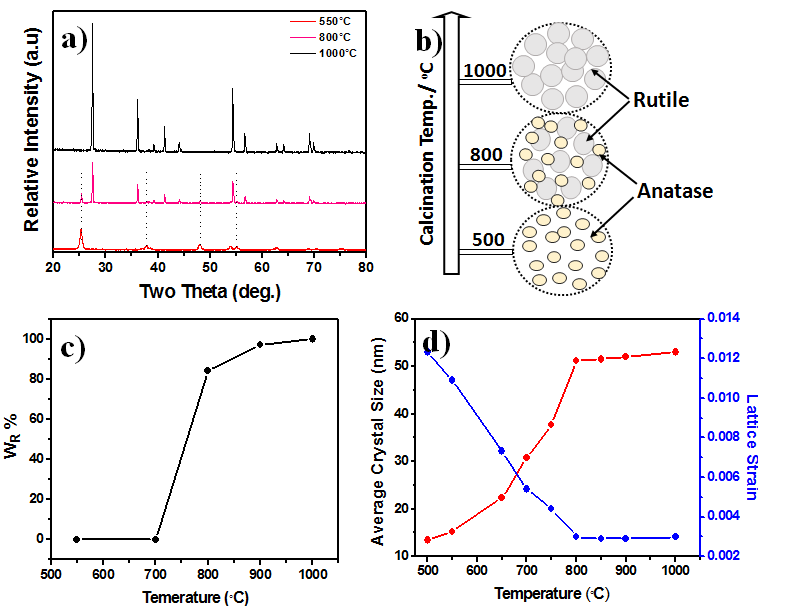


**Figure S1:** a) XRD spectra of anatase TiO_2_ powder at various annealing temperature, b) sketch diagram of annealing temperature influences on particle size, c) Percentage of rutile TiO_2_ at different thermal treatment temperature, and d) Average crystal size-lattice strain of anatase + rutile TiO_2_ along (101+110 ) direction plan *Vs* thermal treatment temperature .


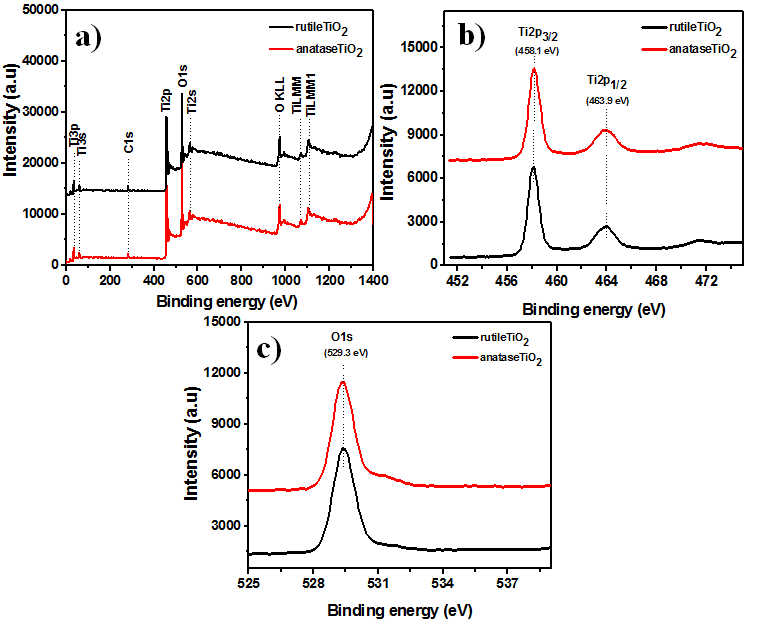


**Figure S2.** XPS analysis of the as-prepared rutile and anatase TiO_2_ samples showing (a) the survey spectra, b) the Ti_2p_, and c) the O_1s_ binding energy measurements.

**
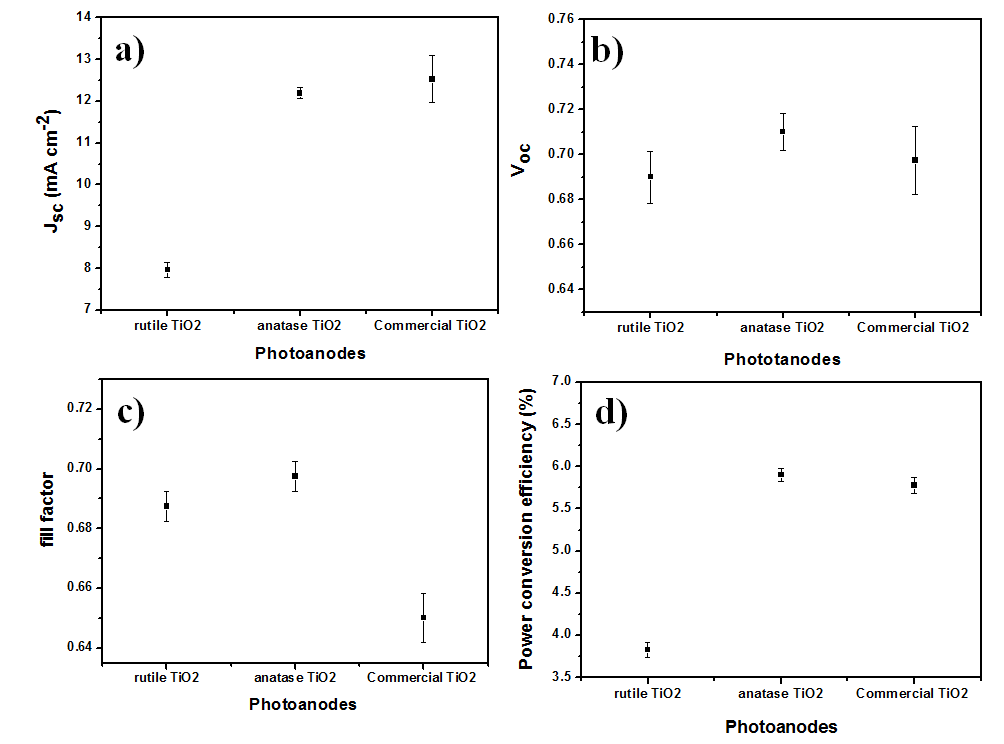
**

**Figure S3** Standard deviation of; a)J_sc_, b)V_oc_, c)ff, and d) of DSSCs fabricated using TiO_2_ nanoparticles

**Table S1:** Hall Measurement and electrical properties of TiO_2_ nanoparticles

| **Photoanode** | **Resistivity**  $\boldsymbol{\times}$ **10^6^ Ω cm** | **Conductivity**  $\boldsymbol{\times}$ **10^-7^ / Ω cm** | **Mobility**  **cm^2^/Vs** | **Sheet Concentration**  $\boldsymbol{\times}$**10^9^ /cm^2^** |
| --- | --- | --- | --- | --- |
| rutile TiO_2_ | 4.18 | 2.39 | 0.039 | -5.67 |
| anatase TiO_2_ | 3.45 | 2.89 | 0.112 | -2.41 |

**Table S2:**  Electronic parameters for fitting Nyquist plots of the various TiO_2_ nanocrystallite photoanodes

| **Photoanode** | **R_S_ (Ω/cm^2^)** | **R_1_(Ω/cm^2^)** | **R_2_(Ω/cm^2^)** | **τ (ms)** |
| --- | --- | --- | --- | --- |
| rutile TiO_2_ | 5.27 | 4.48 | 31.0 | 1.59×10^−4^ |
| anatase TiO_2_ | 5.98 | 2.48 | 19.7 | 2.24×10^−4^ |
| Commercial anatase TiO_2_ | 5.79 | 2.48 | 22.5 | 2.18×10^−4^ |
